# 4029 Stromelysin-1 as a biomarker for acute lung injury

**DOI:** 10.1017/cts.2020.429

**Published:** 2020-07-29

**Authors:** Andrea Sikora Newsome, Rana Kadry, Sandeep Arthram, Timothy Jones, Somanath Shenoy

**Affiliations:** 1 University of Georgia

## Abstract

OBJECTIVES/GOALS: Acute lung Injury (ALI) has long been considered a proceeding event to the development of Acute Respiratory Distress Syndrome (ARDS). Diagnosis of classical ALI and ARDS remains difficult relies on clinical components of the Berlin Criteria, interpretation of radiographs and exclusion of pulmonary edema inducing processes. The precipitating factor for developing ALI involves direct or indirect insult to the lungs. Recent studies have described metalloproteinase-3 (MMP3) to be elevated in plasma samples of patients with lung injury and potentially affected by tobacco use. MMP3 can degrade extracellular matrix components contributing to lung edema and inflammation. This study was conducted to examine the utility of matrix metalloproteinase-3 (MMP3) as a biomarker of lung injury. METHODS/STUDY POPULATION: We conducted a single center, retrospective cohort study of patients admitted to the medical ICU (MICU). De-identified bronchoalveolar fluid (BALF) samples were collected and stored at −80C. Enzymatic activity of MMP3 was determined using a fluorescent resonance energy transfer (FRET) assay. Demographics, comorbidities, evidence of lung injury and patient outcomes were collected. Data were reported with descriptive statistics and data was analyzed with t-tests for statistical significance. RESULTS/ANTICIPATED RESULTS: 55 patient BALF samples were included in the final analysis (mean age 58 +/-17, 58.2% male). 54.5% (n = 30) of patients were determined to have lung injury, 29% (n = 16) of patients had COPD and 45.5% (n = 25) of patients were smokers. MMP3 was higher in patients with lung injury (2363 vs 1052 maxV; p = 0.008). Smoking was associated with decreased MMP3 activity (1231 vs. 2215; p = 0.048). COPD was not associated with differences in MMP3 (1563 vs. 1852; p = 0.605). DISCUSSION/SIGNIFICANCE OF IMPACT: Lung Injury results in elevated MMP3 levels. Smoking was not shown to increase MMP3 levels and may in fact increase them. COPD demonstrated no effect on MMP3 levels. MMP3 levels may vary based on the mode of lung injury (i.e. direct vs indirect) and smoking may impact the activity of the enzyme. Further research should assess activity of MMP3 through different modes of lung injury.

